# Loss of function of *Ywhah* in mice induces deafness and cochlear outer hair cell's degeneration

**DOI:** 10.1038/cddis.2016.88

**Published:** 2016-04-14

**Authors:** L Buret, B Delprat, C Delettre

**Affiliations:** 1 INSERM U1051, Institute of Neurosciences of Montpellier, Department of Biology and Health Sciences, Montpellier, France; 2 Department of Biology and Health Sciences, University of Montpellier, Montpellier, France

The 14-3-3 proteins form a family of seven highly conserved isoforms with chaperone activity, which bind phosphorylated substrates mostly involved in regulatory and checkpoint pathways. These proteins mostly bind serine/threonine-phosphorylated ligands altering their subcellular localization, stability, phosphorylation state, activity or molecular interactions with other targets, thus controlling cell cycle and many signal transduction pathways.^1^ A primary function of 14-3-3 proteins is the inhibition of apoptosis by retaining proapoptotic factors like Bad or Bax in the cytoplasm.^2^ 14-3-3 proteins were originally discovered as abundant molecules in the brain,^3^ comprising about 1% of total proteins of the brain. In the peripheral nervous system, proteomics experiments showed that 14-3-3 proteins were expressed in the cochlea^4^ and among the seven 14-3-3 isoforms, 14-3-3eta encoded by the *YWHAH* gene has been found highly expressed in retinal ganglion cells.^5^ The crucial involvement of 14-3-3 proteins in neuronal physiology led to investigate them in the pathophysiology of neurological diseases as well as considering the *YWHAx* genes as candidate genes in neurodegenerative conditions. Subsequently, 14-3-3 proteins involvement was confirmed in a number of neurological disorders including Parkinson's disease, amyotrophic lateral sclerosis, Alzheimer's disease, epilepsy and Creutzfeldt–Jakob disease,^6^ but whether it is involved in sensory organ dysfunction or degeneration remains unknown. In a recent study published in *Cell Death and Discovery*, we have investigated the function of 14-3-3eta in both auditory and visual systems and we report that the loss of 14-3-3eta protein is associated with cochlear hair cell degeneration and deafness^7^ (Figure 1).

In this study, we showed that the absence of 14-3-3eta in mice causes a hearing loss with a cochlear outer hair cell (OHC) degeneration, only for high frequencies, that is, from 30 to 90 kHz. The auditory exploration of 14-3-3eta-mutant mouse revealed an early and non-progressing decrease of the ABRs suggesting a congenital impairment of hearing in this model. All mutant animals had a significant and stable 15–20 dB threshold shift compared with 14-3-3eta wild type. Distortion product oto-acoustic emissions, which reflect the nonlinear amplification of the OHCs on the basilar membrane motion, were also decreased in mutants.^7^ Buret *et al.*^7^ show that a prominent finding observed in the 14-3-3eta-mutant mice was the abrupt occurrence of massive outer and inner hair cell loss in the basal part of the cochlea. We demonstrated that loss of hair cells in the basal part of 14-3-3eta-mutant cochleas are related to apoptosis and highlight the role of 14-3-3eta in inner ear cell survival. These results were consistent with the notion that apoptosis is a major cause of hearing loss in mammals.^8^

Considering the impact of the loss of 14-3-3eta on the survival of hair cells, we investigated if this protein could lead to auditory diseases. *YWHAH* has been extensively screened in large cohorts of patients (>2000 cases) and controls (>1200 cases) because the *YWHAH* chromosomal location at 22q12.3 co-localizes with the susceptibility loci identified in schizophrenia, bipolar disorders and Parkinson's disease.^9, 10^ However, non-synonymous variants were never identified. By screening *YWHAH* gene in nonsyndromic and syndromic deafness, we reported seven non-synonymous variations never previously found in this gene. To define if these variants could impair the 14-3-3eta function, we have used a combination of two models: downregulation of 14-3-3eta with patient fibroblasts that act for a condition of haploinsufficiency and overexpression of 14-3-3eta mutants in wild-type HeLa cells. We showed that these variants confer an increased susceptibility to apoptosis in fibroblasts, which we were able to reproduce by expressing 14-3-3eta variants in a heterologous system. This deregulated cell death control was related to impair Bad binding to 14-3-3eta. This latter observation further correlates with the alteration of the mitochondrial network fusion process observed in fibroblasts, which is known to be controlled by pro and antiapoptotic members of the Bcl-2 family. As 14-3-3 molecules can interact with a wide variety of cellular proteins, it is possible that genetic variants affect the protection against apoptosis.

These results support the notion that Bcl-2 family proteins such as Bax and Bad are critical for the maintenance of hearing function.^11^ Further data suggest a critical role of this apoptotic pathway in inner hair cell survival, as mutations in the proapoptotic protein SMAC/DIABLO were reported in human-dominant hearing loss, DFNA64^12^ and the proapoptotic Bak protein is involved in age-related hearing loss in mice.^13^ In the cochlea, the death of OHCs after an acoustic trauma has been linked to the activation of proapoptotic Bad.^14^ Altogether, these studies suggest that Bad is a crucial determinant of cell fate in cochlea. Therefore, the defects caused by 14-3-3eta dysfunction might result in Bad activation leading to or facilitating the degeneration of cells that specifically rely on high *YWHAH* expression (Figure 1).

This study shows the essential role of 14-3-3eta in OHC survival. In addition, we report for the first time *YWHAH* variants that can significantly alter the antiapoptotic function of 14-3-3eta by abolishing its interaction with Bad. Altogether, our results underline the fundamental role of 14-3-3eta in auditory physiology.

## Figures and Tables

**Figure 1:**
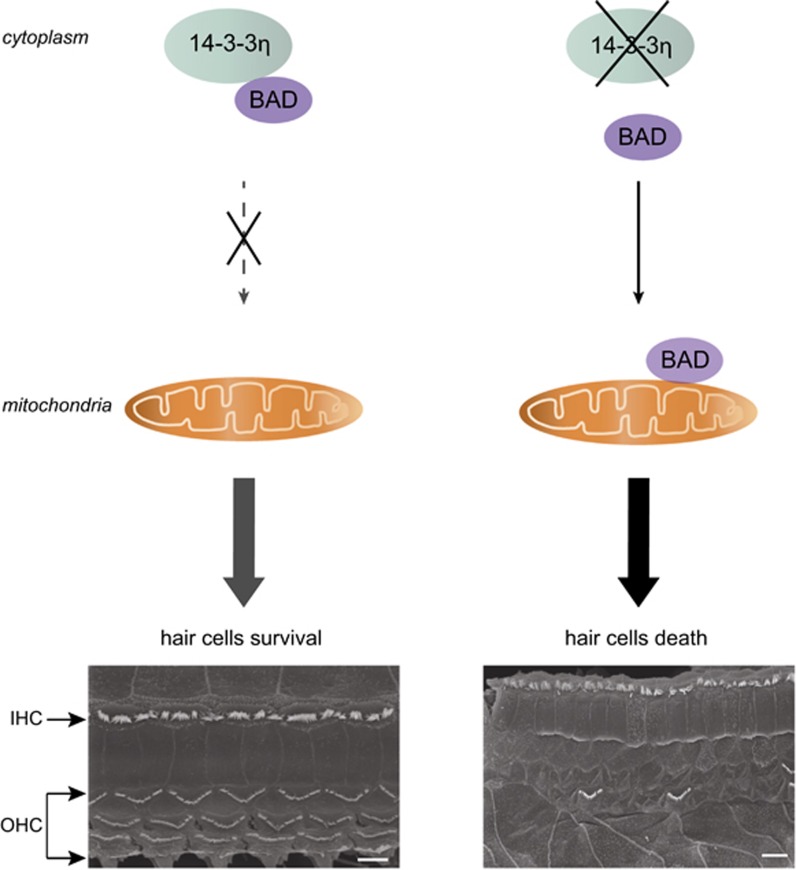
Impact of loss of 14-3-3eta on hair cell death. By controlling the subcellular localization and function of Bad, 14-3-3eta proteins constitute an important regulatory pathway of hair cell death signaling during deafness. Although Bad normally remains sequestered in the cytoplasm by 14-3-3eta scaffold to promote hair cell survival, the loss of 14-3-3eta leads to translocation of this proapototic protein to mitochondria and cell death. Findings of this study support the essential role of 14-3-3eta in hair cell survival and in auditory physiology. IHC, inner hair cells. Scale bars: 5 μm
